# The Properties of Sintered Calcium Phosphate with [Ca]/[P] = 1.50

**DOI:** 10.3390/ijms131013569

**Published:** 2012-10-22

**Authors:** I-Ming Hung, Wei-Jen Shih, Min-Hsiung Hon, Moo-Chin Wang

**Affiliations:** 1 Yuan Ze Fuel Cell Center, Department of Chemical Engineering and Materials Science, Yuan Ze University, No. 135, Yuan-Tung Road, Chungli, Taoyuan 320, Taiwan; E-Mail: imhung@saturn.yzu.edu.tw; 2 Metal Industries Research and Development Centre, 1001 Kaonan Highway, Kaohsiung 81160, Taiwan; E-Mail: petershih@mail.mirdc.org.tw; 3 Department of Materials Science and Engineering, National Cheng Kung University, 1 Ta–Hsueh Road, Tainan 70101, Taiwan; E-Mail: mhhon@mail.ncku.edu.tw; 4 Department of Fragrance and Cosmetic Science, Kaohsiung Medical University, 100 Shih-Chuan 1st Road, Kaohsiung 80782, Taiwan

**Keywords:** sintered, calcium phosphate, hydroxyapatite, density, hardness, microstructure

## Abstract

In order to obtain the properties of the sintered as-dried calcium phosphate with [Ca]/[P] = 1.50, the characteristics of sintered pellets have been investigated using X-ray diffraction (XRD), inductively coupled plasma-mass spectrometry (ICP-MS), Fourier-transform infrared (FT-IR) spectra, Vickers hardness indentation and scanning electron microscopy (SEM). When the pellet samples were sintered between 700 °C and 1200 °C for 4 h, the hydroxyapatite (Ca_10_(PO_4_)_6_(OH)_2_, HA) still maintained the major phase, accompanied with the rhenanite (NaCaPO_4_) as the secondary phase and β-tricalcium phosphate (β-Ca_3_(PO_4_)_2_, β-TCP) as the minor phases. In addition, the HA partially transformed to α-tricalcium phosphate (α-Ca_3_(PO_4_)_2_, α-TCP) and tetracalcium phosphate (Ca_4_(PO4)_2_O, TTCP), when the pellet samples were sintered at 1300 °C and 1400 °C, respectively, for 4 h. The maximum density and Vickers Hardness (HV) of sintered pellet samples were 2.85 g/cm^3^ (90.18% theoretical density (T.D.)) and 407, which appeared at 1200 °C and 900 °C, respectively.

## 1. Introduction

Stoichiometric hydroxyapatite (Ca_10_(PO_4_)_6_(OH)_2_, HA) possesses the excellent properties of a nontoxic, biocompatible, osteoconductive, bioactive, nonimmunogenic agent and it is noninflammatory, *i.e.*, it has the ability to form a direct chemical bond with living tissue as reported by Currey [1]. Dense sintered hydroxyapatite (HA) is very important for tooth root replacement, bone reparation, pulp capping, augmentation of alveolar ridges and maxillofacial reconstruction, *etc.* in clinical application [2]. Fathi *et al.* [3] have pointed out that the recent research trend in the bioceramic field is focused on solving the limitations of calcium phosphates, precisely HA ceramics and in improving their biological properties via exploring the unique advantages of nanotechnology. In addition, due to the fact that the bone mineral crystals are nano-sized with a very large surface area, these crystals are grown in an organic matrix and have very loose crystal-to-crystal bonds leading to homogeneous resorption by osteoclasts [3]. However, the micron size of HA presents a low surface area and has strong crystal-to-crystal bonds. In addition, bone mineral also presents a higher bioactivity compared with synthetic hydroxyapatite [4]. Due to the fact that large-scale bone defects are difficult to restore by autologous bone which is attributed to the extraction of large amounts of autologous tissue leading to problems on the donor’s side such as morbidity or iliac wing fractures [5–7]. In order to solve the problem of those defects, the solid implants consisting of synthetic materials for reconstructing bone and supporting it mechanically, products such as bio-substitute (or bone substitute) implants [8] are used.

In order to enhance the mechanical properties of sintered HA, the properties of the precursor powders have been studied by controlling the important parameters, such as particle size and shape, particle size distribution and agglomeration [9,10]. Engineering HA in the submicron to nanometer range gives it amazing functional properties due to its small crystal size, large surface area to volume ratio, and ultrafine structure similar to biological apatite, which has a great impact on the implant-cell interaction in a body environment. Furthermore, osteoconductivity, solubility, and mechanical reliability of HA can be promoted by controlling its particle size and structural morphology on the nanoscale [11–14].

Many methods for synthesizing HA have been reported, such as sol-gel process [3,15], wet synthesis [10], and the hydrothermal method [16,17]. Using wet processes for preparing HA powders usually results in a fine crystallite size, even submicrons to nanocrystalites, which are better accepted by the host tissue [18,19]. The submicron range of HA powders exhibits a large surface area that can lead to improved sinterability and enhanced densification. Densification of HA is usually achieved via compaction and sintering. The sintering temperature is an important factor that adversely affects the strength of HA [20]. The functional group OH^−^ in the HA matrix can be eliminated by dehydration at a high sintering temperature, but this also causes some HA decomposed leading to formation of α-tricalcium phosphate (α-Ca_3_(PO_4_)_2_, α-TCP), β-tricalcium phosphate (β-Ca_3_(PO_4_)_2_, β-TCP) and tetracalcium phosphate (Ca_4_(PO_4_)_2_O, TTCP) as reported by Zhou *et al.* [21]. In addition, Wang and Chaki [22] also pointed out that when HA was sintered in air for 4 h, the density, Knoop hardness and flexural strength of sintered bodies increased with sintering temperature, reaching a maximum at around 1150 °C and then decreasing because of the decomposition of HA into TCP and TTCP. On the other hand, Kameshet *et al.* [13] also pointed out that the stoichiometric HA sintered between 1000 °C and 1300 °C indicated that no secondary phases such as TCP were present in the HA lattice. The high temperature phase stability of the stoichiometric HA was obtained by sintering at a higher temperature of 1350 °C. In order to understand the sintering ability and phase stability of non-stoichiometric HA, a ratio of [Ca]/[P] = 1.50 work was chosen in the present study. This value is small than the stoichiometric value of 1.67 but near this value. Moreover, the properties of sintered calcium phosphate powders with [Ca]/[P] = 1.50 were studied in detail.

In the present study, the properties of sintering as-dried calcium phosphate powders with [Ca]/[P] = 1.50 have been investigated using inductively coupled plasma-mass spectrometry (ICP-MS), X-ray diffraction (XRD), Fourier-transform infrared (FT-IR) spectra, Vickers hardness indentation and scanning electron microscopy (SEM). The objects of the present study are: (i) to evaluate the phase stability of as-dried calcium phosphate powders with [Ca]/[P] = 1.50 and self accompanying Na_2_CaPO_4_ after sintering, (ii) to evaluate the density and hardness of as-dried calcium phosphate powders with [Ca]/[P] = 1.50 and self accompanying NaCaPO_4_ after sintering and (iii) to observe the microstructure of as-dried calcium phosphate powders with [Ca]/[P] = 1.50 and self accompanying NaCaPO_4_ after sintering.

## 2. Results and Discussion

### 2.1. Phases of As-Dried Calcium Phosphate Powders with [Ca]/[P] = 1.50 after Sintering

The element composition of as-dried calcium phosphate powders determined by ICP-MS analysis is listed in Table 1. This ratio shows that calcium deficient hydroxyapatite was obtained [14]. However, this value is very close to the starting design. Therefore, in the present study, [Ca]/[P] is denoted as 1.50.

**Figure 1:**
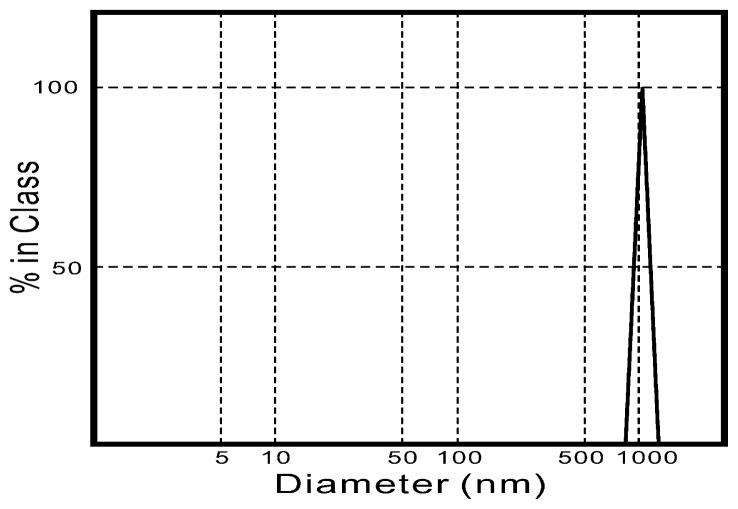
The size distribution of the as-dried calcium phosphate powders with [Ca]/[P] = 1.50.

Figure 1 shows the particle distribution of the as-dried calcium phosphate powders. It can be seen that the distribution was between 0.84 μm and 1.2 μm. Moreover, Figure 1 also shows that the particle size was concentrated at 1.0 μm. This phenomenon due to the fact that as-dried HA powders were synthesized by the wet-chemical routes, where agglomeration can occur during the drying step.

**Figure 2:**
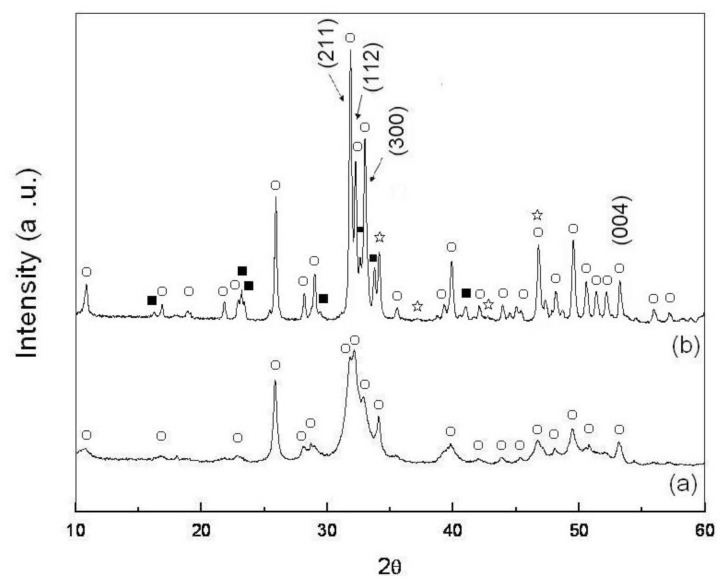
The X-ray diffraction (XRD) pattern of pellet sample of calcium phosphate powders with [Ca]/[P] = 1.50. (**a**) As-dried state and (**b**) Sintered at 600 °C for 4 h (○: HA, ■: NaCaPO_4_, ⋆: β-Ca_3_(PO_4_)_2_).

Figure 2a shows the XRD pattern of the as-dried calcium phosphate powders with [Ca]/[P] = 1.50. It can be seen that there is only a single phase of HA. In addition, boarding and weak intensity of the HA reflections is also seen in Figure 2a, which are due to the poor crystallinity and/or the crystallite size of the as-dried calcium phosphate powders being in the submicron to nanometer scale [23].

The XRD pattern of the pellet sample of the as-dried calcium phosphate powders with [Ca]/[P] = 1.50 was sintered at 600 °C for 4 h as shown in Figure 2b. It reveals hydroxyapatite (Ca_10_(PO_4_)_6_(OH)_2_, HA) (JCPDS Card No. 9-432) as the major phases with the rehenanite (NaCaPO_4_, JCPDS Card No. 76-1456) as the secondary phase and β-TCP (JCPDS Card No. 86-1585) as the minor phase. The peak positions (2*θ*) at 31.90, 32.26, 32.98 and 53.24 correspond to the (211), (112), (300) and (004) reflections of HA, respectively. However, the split of (211) and (112) reflections is not complete due to the fact that HA is not fully crystallized from the pellet sample of as-dried calcium phosphate powders when sintered at 600 °C for 4 h.

Fathi *et al.* [3] used P_2_O_5_ and Ca(NO_3_)_2_ · 4H_2_O as starting materials to synthesize the HA nanopowders and pointed out that a poor crystalline nature of the HA crystal structure with broad diffracted peaks was obtained. Moreover, the phase of CaO was observed when sintered between 750 and 900 °C. These results do not agree with the results in the present study. Fathi *et al.* [3] also pointed out that the additional crystalline phase of TCP appeared when sintering between 700 and 900 °C due to HA decomposition. This result is in agreement with the present work.

**Figure 3:**
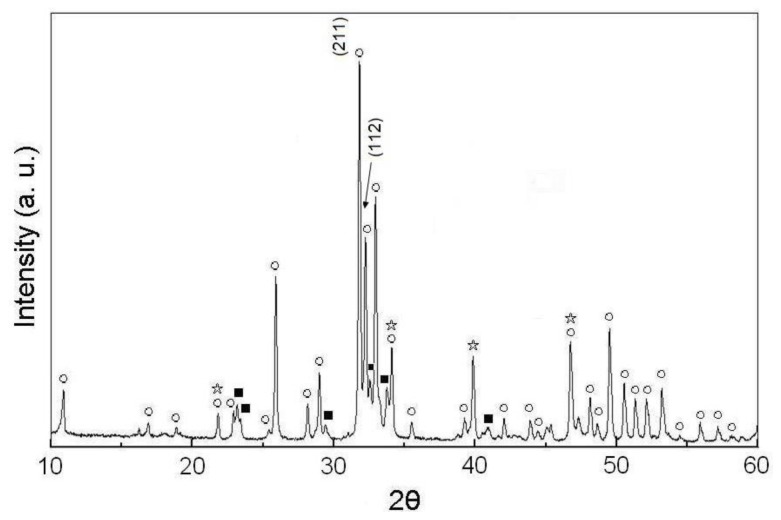
XRD pattern of the pellet sample of as-dried calcium phosphate powders with [Ca]/[P] = 1.50 sintered at 700 °C for 4 h (○: HA, ■: NaCaPO_4_, ⋆: β-Ca_3_(PO_4_)_2_).

Figure 3 illustrates the XRD pattern of the pellet sample of as-dried calcium phosphate powders as sintered at 700 °C for 4 h. It can be seen that while HA still is maintained as the major phase, NaCaPO_4_ and β-TCP are found to as the minor phases. Moreover, although the split of (211) and (112) reflections is clearly more than that found when sintered at 600 °C, it is still not complete. This phenomenon is due to the fact that the atoms in the pellet sample of calcium phosphate powders do not obtain sufficient energy for atom rearrangement at the HA lattice to induce the incomplete crystallization of HA.

**Figure 4:**
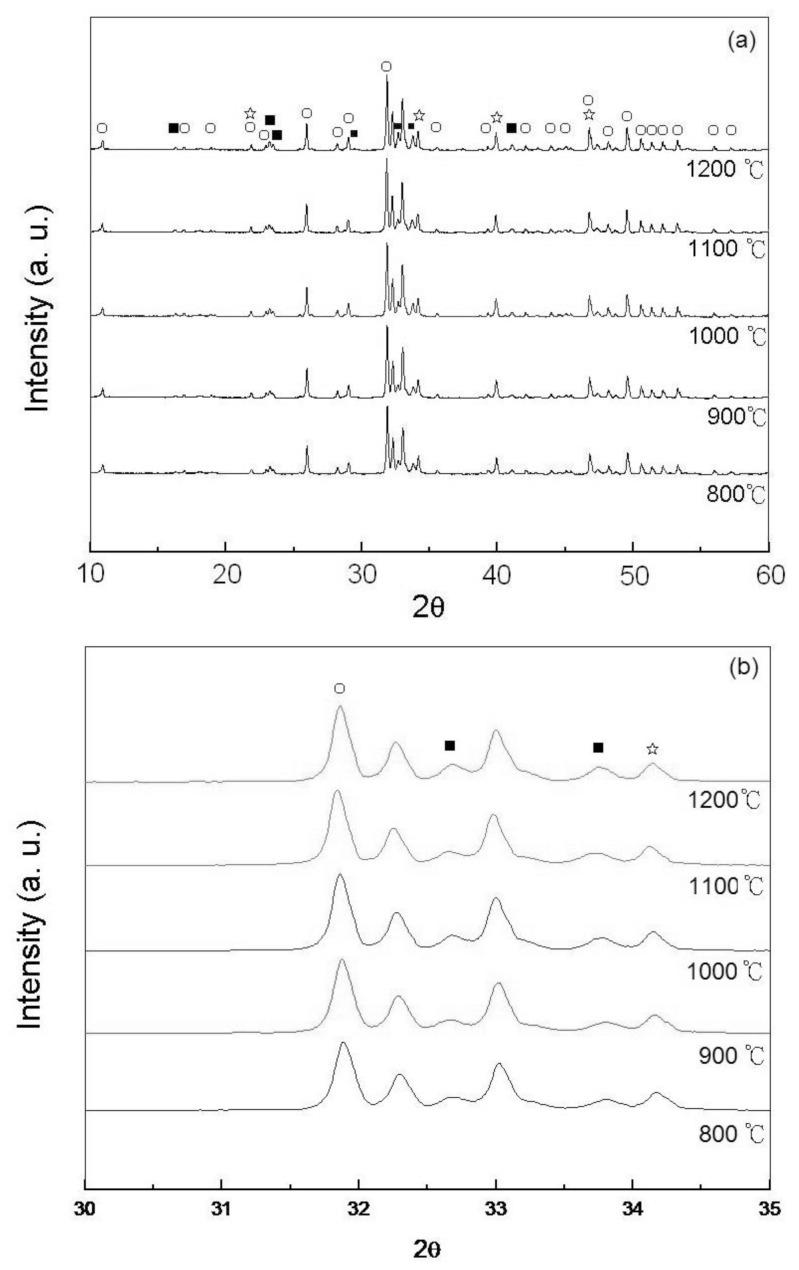
(**a**) XRD patterns of pellet samples of as-dried calcium phosphate powders with [Ca]/[P] = 1.50 sintered between 800 °C and 1200 °C for 4 h and (**b**) zoom on the 30–35° (2*θ*) area (○: HA, ■: NaCaPO_4_, ⋆: β-Ca_3_(PO_4_)_2_).

Figure 4a shows the XRD patterns of the pellet samples of as-dried calcium phosphate powders with [Ca]/[P] = 1.50 sintered between 800 °C and 1200 °C for 4 h. It can be seen that the HA is still maintained as the major phase, and the NaCaPO_4_ and β-TCP are the minor phases. Moreover, the split of (211) and (112) reflections of HA becomes complete at the sintered temperature. This result is attributed to the fact that sintering at a temperature higher than 1000 °C for 4 h only increases the lattice of HA. On the other hand, Figure 4b shows the enlarged view of the Bragg’s (2*θ*) from 30 to 35° of Figure 4a, which reveals the HA as the predominant phase and NaCaPO_4_ phase as the secondary phase for calcium phosphate.

**Figure 5:**
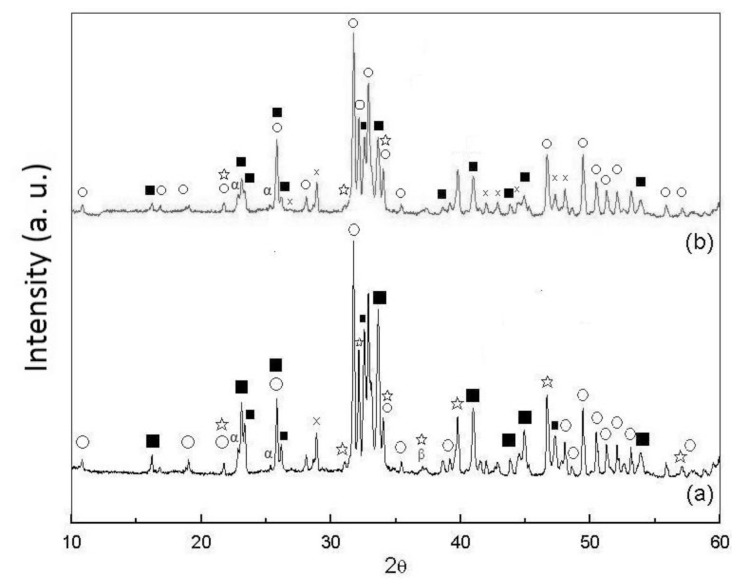
XRD patterns of pellet samples of as-dried calcium phosphate powders with [Ca]/[P] = 1.50 sintered at (**a**) 1300 °C and (**b**) 1400 °C for 4 h (α: α-TCP, ■: NaCaPO_4_, ⋆: β-Ca_3_(PO_4_)_2_, ○: HA, ×: Ca_2_P_2_O_7_).

XRD patterns of the as-dried calcium phosphate powders with [Ca]/[P] = 1.50 sintered at 1300 °C and 1400 °C for 4 h are shown in Figure 5a. It can be seen that when sintered at 1300 °C, the partial HA decomposition leads to the formation of α-tricalcium phosphate (α-Ca_3_(PO_4_)_2_, α-TCP, JCPDS card No. 09-0348), β-TCP and tetracalcium phosphate (Ca_4_(PO_4_)_2_O, TTCP, JCPDS card No. 11-0232). When sintered at 1400 °C, the phases in the XRD pattern are similar to that sintered at 1300 °C.

When the sintering temperature is between 1300 °C and 1400 °C, the HA partially decomposes and transforms into α-TCP, TTCP according to the following reaction [24]:

(1) $${\text{Ca}}_{10}{\left({\text{PO}}_{4}\right)}_{6}{\left(\text{OH}\right)}_{2}\to 2{\text{Ca}}_{3}{\left({\text{PO}}_{4}\right)}_{2}+{\text{Ca}}_{4}{\left({\text{PO}}_{4}\right)}_{2}\text{O}+{\text{H}}_{2}\text{O}$$

Liu *et al.* [23] have denoted the HA decomposition into α-TCP, β-TCP and CaO at 1180–1250 °C. In addition, HA decomposes into β-TCP and CaO when the sintering temperature was increased from 600 °C to 700 °C or higher as reported by Fathi *et al.* [3]. Ramesh *et al.* [13] have pointed out that if HA powders are synthesized with calcium hydroxide and orthophosphate acid as raw materials and the product powders are sintered between 1000 °C and 1300 °C, no secondary phases appear in the HA lattice. Moreover, if HA powders are obtained with H_3_PO_4_ and CaO as raw materials, then HA decomposes into β-TCP when the samples are hot-pressed at 1000 °C for 1 h and sintered at 1200 °C for 2 h, as also reported by Tang *et al.* [14]. Furthermore, Wang and Chaki [22] used Ca(NO_3_)_2_·4H_2_O and (NH_4_)_2_·HPO_4_ as the starting materials for synthesizing HA powders. When these product powders were sintered at 1100 °C for 4 h in air, HA started to decompose into β-TCP. Upon heating to 1300 °C, the peaks of TTCP appear. After sintering at 1350 °C for 4 h, some of the β-TCP converted to α-TCP. Therefore, in the present study, the reaction of Equation 1 occurs at 1300 °C is attributed to various raw materials used and different synthesized process [3,14,22,25].

According to Figures 2–5, the small amount of NaCaPO_4_ appears to be due to the incorporation of Na^+^ in the synthetic HA [26]. Furthermore, in Figures 2–4, the XRD diffraction peaks of HA shift to a higher angle by 0.05–0.13° of 2*θ*, which indicates that the lattice of HA is due to the loss of OH radicals [22,24,27].

From the result shown in Figures 2–5, the phase transformation of as-dried calcium phosphate powders with [Ca]/[P] = 1.50 can be divided to two stages: (i) from 600 °C to 1200 °C as principal stage, and (ii) from 1200 °C as high temperature stage. Moreover, the NaCaPO_4_ first appears at 600 °C and still persists up to 1400 °C.

On the other hand, according to the results of XRD except for the intensity of HA, NaCaPO_4_ and β-Ca_3_(PO_4_)_2_, the other minor phases can be neglected. Then, the volume fraction of the NaCaPO_4_ can be estimated by the following modified formula [28]:

(2) $${\text{NaCaPO}}_{4}\;(\%)=\frac{{\text{I}}_{\text{Na}}\;(611)}{{\text{I}}_{\text{HA}}\;(211)+{\text{I}}_{\text{Na}}\;(611)+{\text{I}}_{\beta }(020)}$$

where *I*_HA_ (211), *I*_Na_ (611), and *I*_β_ (020) refer to the relative intensity of (211), (611) and (020) of the HA, NaCaPO_4_ and β-Ca_3_(PO_4_)_2_ phases, respectively.

According to the results of Figures 2–5 and calculated by Equation 2, the results indicate that the volume fractions of NaCaPO_4_ in the sintered pellets are about (15 ± 1)%.

When the pellet samples of as-dried calcium phosphate powders with [Ca]/[P] = 1.50 were sintered between 600 °C and 1200 °C for 4 h, the results of XRD patterns show HA as the major phase. Ishikawa *et al.* [25] have pointed out that most of this is calcium-deficient hydroxyapatite (d-HA, Ca_10−_*_x_*(HPO_4_)*_x_*(PO_4_)_6−_*_x_*(OH)_2−_*_x_*·xH_2_O, for 0 < *x* ≤ 1) are supposed to decompose into β-TCP and stoichiometric HA (s-HA) with a slow reaction rate over 650 °C and still continue even above 1000 °C. However, according to Figures 2–5, only small amounts of β-TCP appeared when the sintering temperature was lower than 1400 °C. Ardo and Matsuno [29] investigated the Ca_3_(PO_4_)_2_-CaNaPO_4_ system and found that NaCaPO_4_ dissolved a large amount of β-TCP when heated between 650 °C and 980 °C, containing 50.5% to 97% NaCaPO_4_ to form an NaCaPO_4_ solid solution. Therefore, in the phase of β-TCP, only small amounts appeared when the sintering temperature was lower than 1200 °C in the present study and this was due to the formation of a NaCaPO_4_ solid solution, which inhibited the formation of other phases of calcium phosphate.

Wang and Chaki [22] have pointed out that when HA sintered at 1100 °C and 1350 °C in air for 4 h, it started to decompose and convert into β-TCP. The phase of TTCP appeared when sintering at 1300 °C. Some of the β-TCP converted to α-TCP when sintering at 1350 °C. In the present study, the decomposition of HA partially started to convert into α-TCP and TTCP when sintering in air at 1300 °C for 4 h. This result is in agreement with the report of Wang and Chaki [22].

On the other hand, Moseke and Gbureck [30] have pointed out that TTCP is the only calcium phosphate with a [Ca]/[P] ratio greater than 1.67, and it is formed in the system of CaO-P_2_O at temperatures greater than 1300 °C. Moreover, due to the fact that TTCP is metastable, the synthesis of phase-pure TTCP requires either a rapid quench or the absence of moisture to prevent decomposition into HA and lime in the temperature range of 1000–1200 °C [31]. In the present study, the composition of HA partially formed α-TCP, TTCP and H_2_O. The decomposition of TTCP to form HA and CaO, and the reaction of HA decomposing to α-TCP, TTCP and H_2_O, seems to be a thermodynamical process. This result is not in agreement with the report of Moseke and Gbureck [30], but in agreement with the report of Lin *et al.* [24].

### 2.2. FT-IR Spectra of Pellet Samples of As-Dried Calcium Phosphate Powders with [Ca]/[P] = 1.50 as Sintered at Various Temperatures for 4h

**Figure 6:**
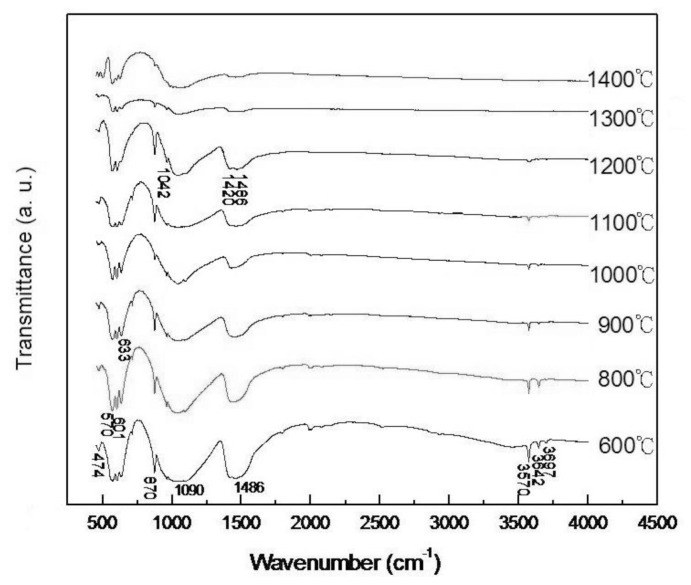
Fourier-transform infrared (FT-IR) spectra of pellet samples of as-dried calcium phosphate powders sintered at various temperatures for 4 h.

Figure 6 shows the FT-IR spectra of pellet samples of as-dried calcium phosphate powders with [Ca]/[P] = 1.50 sintered at various temperatures for 4 h. It can be seen that the spectra at 3697, 3642 and 3570 cm^−1^ are related to the stretching vibrations of the free hydroxyl group and that 630 cm^−1^ is linked to the OH^−^ libration mode [32,33]. There are CO_3_^2−^ ν_3_ bonds at 1420 and 1486 cm^−1^ [33]. In addition, the bands located in the range of 1042 and 1090 cm^−1^ are consistent with phosphate group absorption in HA [33]. Moreover, the band at 873 cm^−1^ indicates the n_2_ mode of a CO_3_^2−^ group for some carbonated HA (Ca_10_(PO_4_)_6_(CO_3_), CHA) formed [33–35] or an HPO_4_ group [3]. The PO_4_^3−^ ν_1_ mode at 980 cm^−1^ reveals the presence of asymmetric HA [35], when sintering between 600°C and 1200 °C. However, when sintering at 1300 °C and 1400 °C, the PO_4_^3−^ ν_1_ mode at 980 cm^−1^ disappears. This result corresponds to the XRD results. Furthermore, it can also be seen that there is the PO_4_^3−^ ν_4_ mode at 570 and 601 cm^−1^, and a PO_4_^3−^ ν_2_ mode at 504 cm^−1^, which reveals an apatite structure.

In addition, the non-apatitic component CO_3_^2−^ of HA has the B-type preferred carbonate composition, which reveals the substitution of OH^−^ (A-site) or PO_4_^3−^ (β-site). The CO_3_^2−^ at 873 cm^−1^ indicates the formation of B-site CHA [36]. This result is attributed to the fact that the CO_2_ in the air and water precipitate at with hydrolysis reaction according to the following equation:

(3) $${\text{CO}}_{2}+2{\text{OH}}^{-}\to {{\text{CO}}_{3}}^{2-}+{\text{H}}_{2}\text{O}$$

Therefore, the higher solubility of CO_3_^2−^ was obtained in the alkaline solution [36].

In Figure 6, it can also be seen that the 3570, 3642 and 3697 absorptions are more or less masked by the absorption of water. The OH absorption band for the sample sintered at 600 °C exhibits sharp peaks, whereas for the sample sintered at 1200 °C there is a reduction in intensity and these absorptions vanish when sintered at 1300 °C and 1400 °C for 4 h. This result reveals that the phase of hydroxyapatite is fully hydroxylated at 600 °C, but it is considerably dehydroxylated at a temperature higher than 600 °C [22]. The absorptions of 3570, 3642, 3697 decrease from 600 °C to 1200 °C due to the dehydroxylated occurring with the rise of the sintering temperature. This result is consistent with the lattice contraction found in the XRD results of Figures 2–4. Whereas the 3570, 3642 and 3697 vanished when sintered at 1300 °C and 1400 °C due to the HA partial decomposition into α-TCP and TTCP. These results coincide with Figure 6. The dehydrated material can be regarded as being composed of oxyhydroxyapatite, Ca_10_(PO_4_)_6_(OH)_2−2_*_x_*O*_x_*□*_x_* (□: vacancy, *x* < 1; OHA) with vacancies located on hydroxyl sites [22,37].

### 2.3. Density and Hardness of Pellet Samples of As-Dried Calcium Phosphate Powders with [Ca]/[P]=1.50 Sintered at Various Temperatures

**Figure 7:**
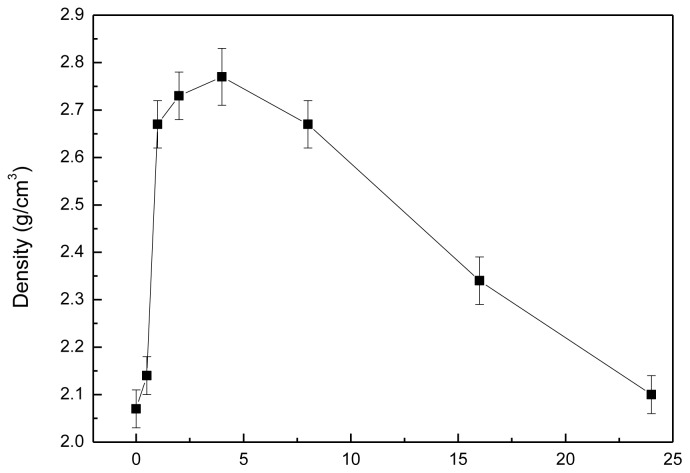
The density of pellet samples of as-dried HA powders with [Ca]/[P] = 1.50 sintered at 800 °C for various durations.

Figure 7 shows the density of the pellet samples of as-dried calcium phosphate powders with [Ca]/[P] = 1.50 sintered at 800 °C for various durations. The results reveal that the density of sintered pellets was 2.07 g/cm^3^, which is only 65.50% of the theoretical density (T.D. 3.16 g/cm^3^ [20]) of HA when sintered at 800 °C for 1 min. The density of sintered pellets rapidly increased from 2.07 to 2.77 g/cm^3^ (87.66% T.D.) when sintered at 800 °C for 4 h. Kalita *et al.* [38] have pointed out that the mechanical properties of HA often improve significantly by adding a small amount of CaO-P_2_O_5_-Na_2_O-based sintering additives without altering its excellent biocompatibility and low sintering temperature. In the present study, although we did not add any sintering additive to the as-dried calcium phosphate powders, the density of sintered pellets rapidly increased with increasing sintering time from 1 min to 4 h because the NaCaPO_4_ formation promoted the sintering ability and enhanced the densification. Therefore, the phase of NaCaPO_4_ may be looked at as the sintering additives in the present study. After sintering at 800 °C from 4 to 24 h, the density of sintered pellets gradually decreased from 2.77 to 2.10 g/cm^3^ (66.46% T.D.). Sintering of HA is complicated by dehydroxylation and decomposition of HA at a higher temperature as reported by Wang and Chaki [22]. When the pellet samples of as-dried calcium phosphate powders were dehydroxylated, HA lost OH radicals upon heating [13] and longer hold times applied according to the following equation [23,27]:

(4) $${\text{Ca}}_{10}{\left({\text{PO}}_{4}\right)}_{6}{\left(\text{OH}\right)}_{2}\to {\text{Ca}}_{10}\;{\left({\text{PO}}_{4}\right)}_{6}\;{\left(\text{OH}\right)}_{2-2x}\;{\text{O}}_{2}{□}_{x}\;+\;{\text{xH}}_{2}\text{O}↑$$

The hydroxyl ion-deficient product of Ca_10_(PO_4_)_6_(OH)_2−2_*_x_*O_2_□*_x_* was known as oxyhydroxy-apatite (OHA). In air, OHA was formed at around 900 °C [22]. In the present study, when sintering at 800 °C for 4 h, the density reached a maximum value, then decreased with sintering lime longer than 4 h. This result is due to the dehydroxylation reaction that rapidly occurred when sintering time was longer than 4 h.

**Figure 8:**
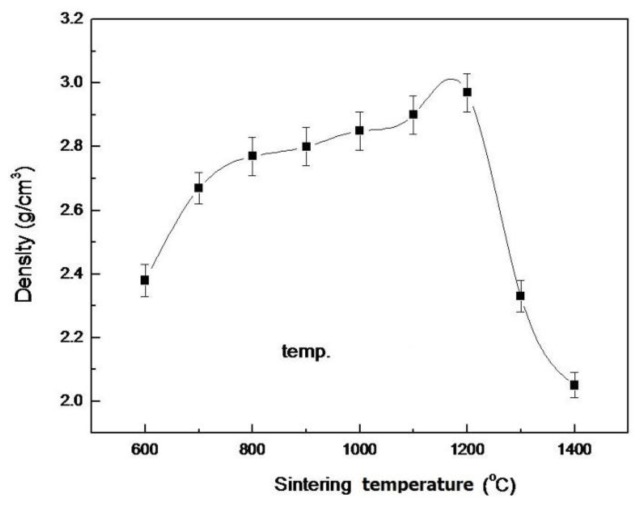
The density of pellet samples with [Ca]/[P] = 1.50 sintered at various temperatures for 4 h.

The density of pellet samples of as-dried calcium phosphate powders with [Ca]/[P] = 1.50 sintered at various temperatures for 4 h is shown in Figure 8. It reveals that the density is only 2.38 g/cm^3^ (72.32% T.D.) for the pellet sample of as-dried calcium phosphate powders with [Ca]/[P] = 1.50 sintered at 600 °C for 4 h. However, the sintering sample has sufficient strength to measure the density in water. The density increases from 2.77 g/cm^3^ to 2.90 g/cm^3^ (91.77% T.D.) when the sintering temperature was increased from 800 °C to 1100 °C. When sintering at 1200 °C, the maximum density of 2.85 g/cm^3^ (90.18% T.D.) was obtained. Then, the density decreased to 2.05 g/cm^3^ (64.87% T.D.) when sintering at 1400 °C for 4 h because HA partially decomposed into α-TCP and TTCP. In addition, the density of 2.85 g/cm^3^ is only 90.33% of T.D., *i.e*., it is still lower than the theoretical density (3.21 g/cm^3^) of NaCaPO_4_ [39].

In the present study, the lower density of pellet samples was obtained when sintered at 600 °C for 4 h due to loss water of HA by dehydroxylation [22]. The density of sintering pellet samples of as-dried calcium phosphate powders with [Ca]/[P] = 1.50 increased from 2.38 to 2.85 g/cm^3^ with rising a sintering temperature from 600 °C to 1200 °C. This result was due to the NaCaPO_4_ increasing with increasing temperature. Moreover, the reaction of dehydroxylation also accompanied the OHA formation with NaCaPO_4_, as the sintering agent OHA has a good sinterability and, therefore, leads to an increased density. On the other hand, according to Figures 2–5, the decomposition of HA started at 1300 °C when sintering the pellet sample of as-dried calcium phosphate powders in air. However, the dehydroxylation of HA in air occurred at temperatures lower than 1300 °C [22] and enhanced the density increase with increasing sintering temperature.

It may be concluded that during sintering of HA, grain growth was accompanied by the creation of many vacancies in the apatite crystallites, and that the sintered product is OHA [40,41]. The increased reactivity in dehydration and the resultant vacancies may enhance sintering [40]. On the other hand, the densification of ceramics in a solid state sintering is a process of pore elimination, accomplished by a diffusion process through the transfer of matter from the particle volume or from the grain boundary between particles [42]. Thus, in the present study, it can be suggested that the transfer of matter from the particle volume was substantially enhanced with the sintering process.

On the other hand, the density decreased rapidly when the pellet samples were sintered at 1300 and 1400 °C due to the partial HA decomposition into α-TCP and TTCP [13,24]. The thermal decomposition was accompanied by the two steps of dehydroxylation and decomposition [43].

**Figure 9:**
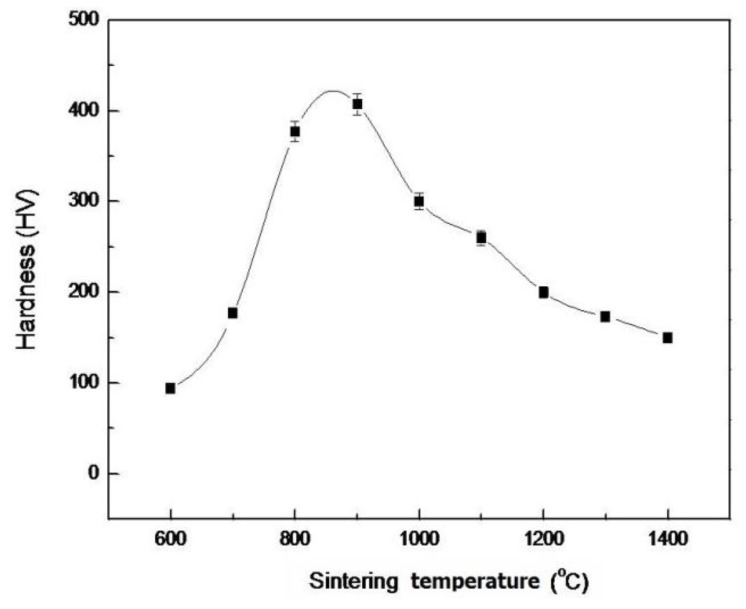
The surface Vickers Hardness (HV) of pellet samples with [Ca]/[P] = 1.50 sintered at various temperatures for 4 h.

Figure 9 shows the surface Vickers Hardness (HV) of pellet samples of as-dried powders with [Ca]/[P] = 1.50 sintered at various temperatures for 4 h. The HV hardness of the sintered HA rapidly increases from 94.0 to 407 when the sintering temperature increases from 600 °C to 900 °C, then rapidly decreases to 150 when sintering at 1400 °C. The maximum hardness of the sintered HA with [Ca]/[P] = 1.50 is present at 900 °C, and is not present at 1200 °C.

The variation of the hardness of sintered pellets with sintering temperatures was not similar to that of density, indicating that the hardness was not only controlled by the bonding among the particles in the sintered compact, but also controlled by other factors such as grain size and secondary phases. The present result of hardness did not agree with the result of Wang and Chaki [22]. On the other hand, the hardness variation of the present study is also not in agreement with the general trend of hardness increase with rising sintering temperature [44,45].

In the present work, it was found that the hardness of sintered pellets decreased with increasing temperature beyond the point at which maximum hardness was obtained. The decreases in hardness observed for pellet samples of as-dried calcium phosphate powders with [Ca]/[P] = 1.50 sintered above 900 °C (*i.e.*, from 1000 to 1400 °C) could not be due to density effect but rather could be caused by the grain growth of HA.

### 2.4. SEM Microstructure of Pellet Samples of As-Dried Calcium Phosphate Powders after Sintering

**Figure 10:**
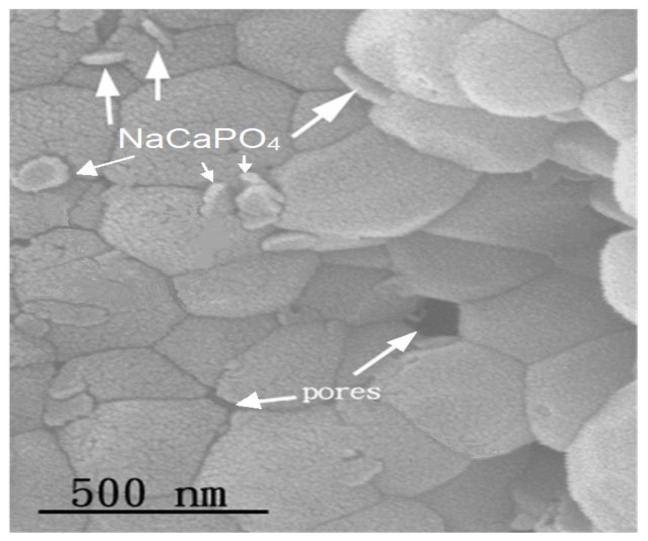
Scanning electron microscopy (SEM) image of the as-dried calcium phosphate powders with [Ca]/[P] = 1.50 sintered at 800 °C for 24 h. The NaCaPO_4_ and pores are indicated by arrows.

Figure 10 shows the pellet samples of as-dried calcium phosphate powders with [Ca]/[P] = 1.50 sintered at 800 °C for 24 h. A polygonal grain with a size of about 250–500 nm was observed. This result, due to the required energy for grain growth of HA, was obtained when sintering at 800 °C for a prolonged period of time. In addition, the NaCaPO_4_ (indicated by arrows) precipitated between the grain boundaries of HA, and pores were also observed.

**Figure 11:**
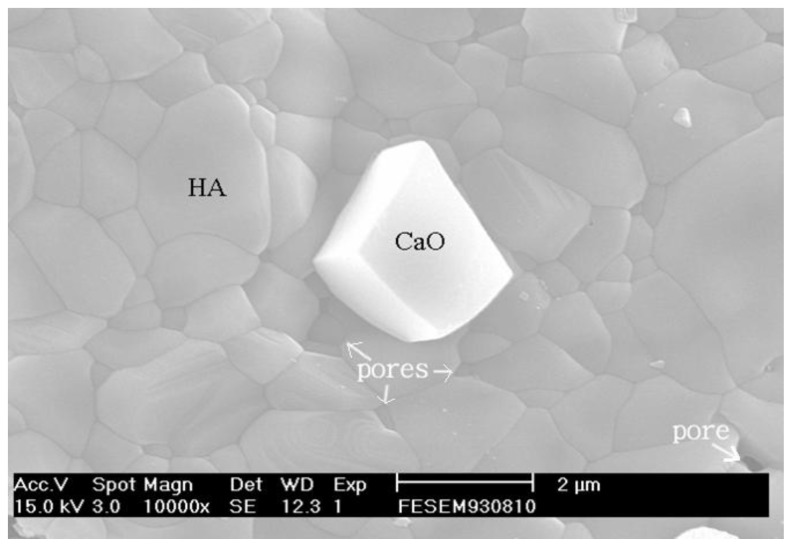
SEM image of a calcium phosphate pellet with [Ca]/[P] = 1.50 sintered at 1000 °C for 4 h.

Figure 11 shows the pellet sample of as-dried calcium phosphate powders with [Ca]/[P] = 1.50 sintered at 1000 °C for 4 h. It can be seen that HA grains of a size of about 0.6–2.0 μm are observed, as well as CaO and residual pores.

Considering Figures 10 and 11, it is interesting to note that the rate of grain growth is very low when sintering at 800 °C. This is very important, as materials with a small grain size would normally result in enhanced mechanical properties when compared to the same materials having a larger grain size.

As mentioned above, the effects on the calcium phosphate pellets with [Ca]/[P] = 1.5 sintered between 700 and 1400 °C lead to a HA which possesses a good strength and hardness. In the present contribution, HA was obtained from the calcium phosphate powders with [Ca]/[P] = 1.5 prepared by hydrolysis of hydrogen phosphate dehydrate (CaHPO_4_·2H_2_O, DCPD). This result also reveals that in order to obtain the HA it is not necessary for the calcium phosphate to have the exact value of [Ca]/[P] = 1.67. With calcium phosphate powders with values of [Ca]/[P] smaller than 1.67, the HA structure is also obtained.

## 3. Experimental Section

### 3.1. Sample Preparation

The starting materials were hydrogen phosphate dehydrate (CaHPO_4_·2H_2_O, DCPD, purity ≥98.0%, supplied by Riedel-de Haën, Germany) and CaCO_3_ (purity ≥98.5%, supplied by Riedel-de Haën, Germany). DCPD and CaCO_3_ were dissolved in a deionized water and ethanol solution at a volume ratio of 1:5. The concentrations of DCPD and CaCO_3_ were 0.5 M. The ratio of [Ca]/[P] was designed as 1.5 for the synthesis process. The 0.5 M solution was 500 mL of 2.5 M NaOH solution (pH = 13.6, purity ≥90.0%, supplied by Showa, Japan), with CaCO_3_ added. Both mixtures were blended in an agitator with a speed of 200 revolutions per minute (rpm). When hydrolysis at 75 °C for 1 h attained the pH value of 11.0, then the reaction was terminated by cooling in ice water. The aggregates were filtered and then rinsed in deionized water five times. Subsequently, the product of calcium phosphate was dried at 60 °C for 12 h. Then, the as-dried calcium phosphate powders were obtained. In order to prevent the occurrence of serious agglomeration, the as-dried powders must be thoroughly. The as-dried powders were put in a laboratory ball mill using Al_2_O_3_ balls for 6 h. Then, the ground powders were put through an 80-mesh sieve.

The as-dried calcium phosphate powders were prepared in our laboratory. The fine powders were mechanically blended with 1 wt% PVA binder in a high-shear mill for 2 h and sieved after granulation. Each sample used 0.25 g as-dried calcium phosphate powders that were uniaxially cold-pressed at 23 MPa to form a pellet with a diameter of 10 mm.

Finally, some pellet samples were put in a sealed aluminum crucible and sintered at 800 °C for 1 min to 24 h, with a heating rate of 3 °C/min. Subsequently, the sintering pellet was cooled to room temperature at a rate of 5 °C/min. Moreover, other pellets were sintered between 600 °C and 1400 °C with a heating rate of 3 °C/min to each predetermined temperature for 4 h. Then, they were furnace cooled to room temperature at a rate of 5 °C/min. Each sintering condition was repeated five times, then the sintered samples for every condition were obtained.

### 3.2. Sample Characterization

The concentration of elements and ratio value of [Ca]/[P] detected by inductively coupled plasma-mass spectrometry (ICP-MS, ELAM 6100 DRC II ICP-MS Perkin-Elmer, Concord, ON, Canada) was used for these experiments and dissolved in 50% concentrated *aqua regia* solution. Samples were introduced by a pneumatic nebulizer with a Scott type spray chamber. The operating conditions of ICP-MS were optimized by continuous introduction of solution after the powders had dissolved. The pure water was treated as the blank and was added as standard solution for the calibration curve. The solution flow rate was maintained at about 1.5 mL/min.

The particle size distribution of as-dried calcium phosphate powders was measured by using an ultrafine particle analyzer with a 6328 Å He-Ne laser as a detecting source (Zetasizer 2000, Malvern Instrument).

The chemical behavior and molecular bonding structure of the as-dried calcium phosphate powders sintering at various temperatures for 4 h were evaluated using a Fourier-transform infrared spectroscope (PerkinElmer spectrum One FT-IR spectrometer, Boston, MA, USA). Each sintered pellet sample was reground to powders. Each sample was mixed with KBr (sample: KBr = 1:99 in mass ratio) and was pressed into 200 mg pellets, 13 mm in diameter, and the infrared adsorption spectra were measured in a frequency range of 400 cm^−1^ to 4000 cm^−1^. A spectral resolution of 4 cm^−1^ was chosen, and the composite spectrum for each sample was represented by the average of 64 scans, normalized to the spectrum of the blank KBr pellets.

The surface of sintered pellet samples was ground with Al_2_O_3_ powders from #100 to #1200 with H_2_O. Subsequently, polished by diamond paste for X-ray diffraction (XRD), density measurement and hardness test.

Identification of the crystalline phases in the sintered pellets was carried out by XRD analysis (Models D-Max/IIIV, Rigaku, Tokyo, Japan) with Cu Kα radiation and Ni filter, operated at 40 kV, 30 mA, and a scanning rate (2*θ*) of 1°/min from 20 to 55°. Then, the XRD patterns were obtained for the polished bulk surface of the sintered pellets.

The apparent density (ρ) of the sintered pellet was measured by the Archimedes method using a sample size of 4.0 × 4.0 × 8.5 mm^3^ and calculated as follows:

(5) $$\rho =\frac{w_{\text{a}\times \text{s}}}{w_{\text{a}}-w_{\text{b}}}$$

where *w*_a_ is the weight of a dried sample in air (g), *w*_b_ is the weight of a sample in deionized water (g) and *s* is a specific gravity of a deionized water (g/cm^3^). When the sample weight in deionized water, the suspension wire of copper with diameter 0.12 mm may be twisted around the test specimen.

The hardness was measured by a Vickers indentation hardness tester (INDENTAMET 1100 SERIES, RUEHLER, USA). During Vickers indentation, the load applied and holding time on the polished surface of sintered pellet were 1.96 N and 30 s, respectively. In each sample, 10 indentations were measured. For scanning electron microscopy (SEM) observation, the polished sintered pellets were etched with dilute acetic acid. The operated conditions of gold-coating were 110 V, 10 mA and 2 min for voltage, current and time, respectively, to obtain the required film thickness for observation. The microstructure was observed by SEM (Model XL40 FE-SEM, Philips, Eindhoven, The Netherlands).

## 4. Conclusion

The properties of sintering as-dried calcium phosphate powders with [Ca]/[P] = 1.50 have been investigated using ICP-MS, XRD, FTIR, Vickers hardness test and SEM. The as-dried calcium phosphate powders have a single phase of HA when sintered between 600 and 1200 °C, whereby HA and NaCaPO_4_ appeared as the major and second phases, respectively, with β-TCP as the minor phase. When the pellet samples were sintering at 1300 °C and 1400 °C, the HA partially decomposited into α-TCP and TTCP. The result of the FTIR showed that the dehydroxylation of HA occurred when sintered between 600 °C and 1200 °C. The density of sintering pellets increased from 2.38 to 2.85 g/cm^3^ (72.32 to 90.18% T.D.) with rising temperatures from 600 to 1200 °C due to the NaCaPO_4_ and oxyhydroxyapatite (OHA) formed with the temperature increase and the enhancement of densification. Whereas the density abruptly decreased to 2.05 g/cm^3^ (64.98% T.D.) when sintered at 1400 °C for 4 h due to partial decomposition of HA into α-TCP and TTCP. In addition, the maximum HV hardness of sintered pellets appeared at 900 °C, then decreased with rising sintering temperatures. When the pellet was sintered at 800 °C for 24 h, polygonization HA grains of a size of about 250–500 nm was observed. When sintering at 1000 °C for 4 h, HA grains of a size of about 0.6–2.0 μm were obtained.

## Figures and Tables

**Table 1:** The element composition of as-dried calcium phosphate powders.

Element	Concentration (mg/L)	Concentration (mmol/L)
Na	57	2.5
Ca	3971	99.27
P	2026	67.53
